# An Unusual Cause of Vesical Calculi

**DOI:** 10.7759/cureus.6701

**Published:** 2020-01-19

**Authors:** Arunkumar Jamburaj, Danny Darlington, Jagatheswaran Chinnathambi

**Affiliations:** 1 Urology, Pondicherry Institute of Medical Sciences, Pondicherry, IND

**Keywords:** encrustation, migration, vesical calculus, copper intrauterine contraceptive devices

## Abstract

Intravesical migration is an uncommon but serious complication of intrauterine contraceptive devices. Calculus formation is common over such migrated intrauterine contraceptive devices. This dreaded complication usually presents with lower urinary tract symptoms such as suprapubic pain, frequency, and nocturia. We present a case of a 50-year-old woman with intravesical migration of copper-T device placed in the immediate postpartum period 25 years ago. She presented with dysuria, which was confirmed by computed tomography. The migrated device was encrusted with a 3.5-cm-sized stone around its vertical limb. Another stone of approximately the same size was present in the bladder. Surprisingly, the patient never had symptoms and hence she never followed up for 25 years. The stones could not be removed endoscopically, and therefore an open vesicolithotomy was performed. This case has been presented to highlight the significance of following up patients with intrauterine contraceptive devices to avoid potentially devastating complications.

## Introduction

Intrauterine contraceptive devices (IUCDs) are widely employed to achieve reversible contraception. They are cost-effective and have low complication rates. Rarely, uterine perforation can occur during copper IUCDs (Cu-IUCD) insertion in about 1.6 per 1,000 insertions [1]. However, intravesical migration and stone formation are extremely rare, as, usually, the patient presents early because of the persistent lower urinary tract symptoms (LUTSs) once intravesical migration occurs. We report a case of a 50-year-old woman who presented with asymptomatic stone formation over a Cu-IUCD, which migrated into the bladder. Surprisingly, the patient was asymptomatic for 25 years, which explains the delay in the diagnosis and treatment. This case represents the longest period of forgotten and migrated Cu-IUCD with calculus formation reported in the literature.

## Case presentation

**Figure 1:**
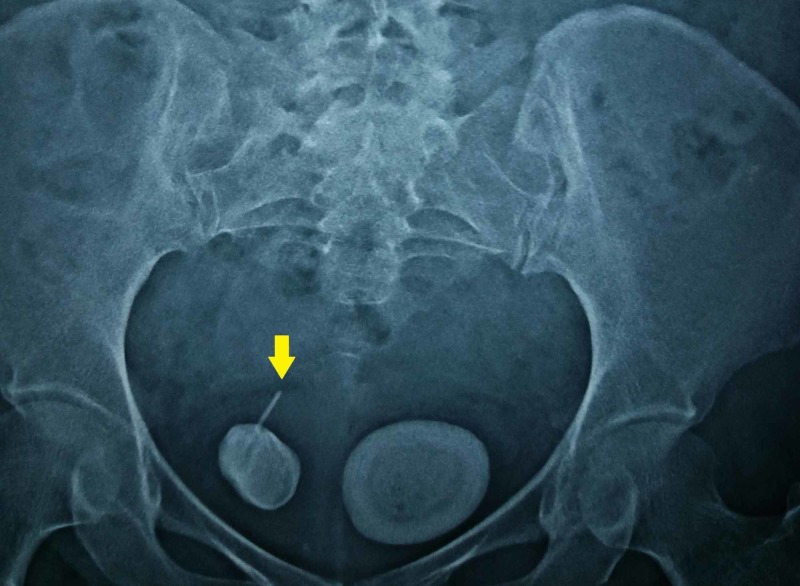
X-ray image of the kidney, ureter, and bladder region showing the presence of two vesical calculi with a linear radio-opaque extension suspicious of foreign body (yellow arrow)

**Figure 2:**
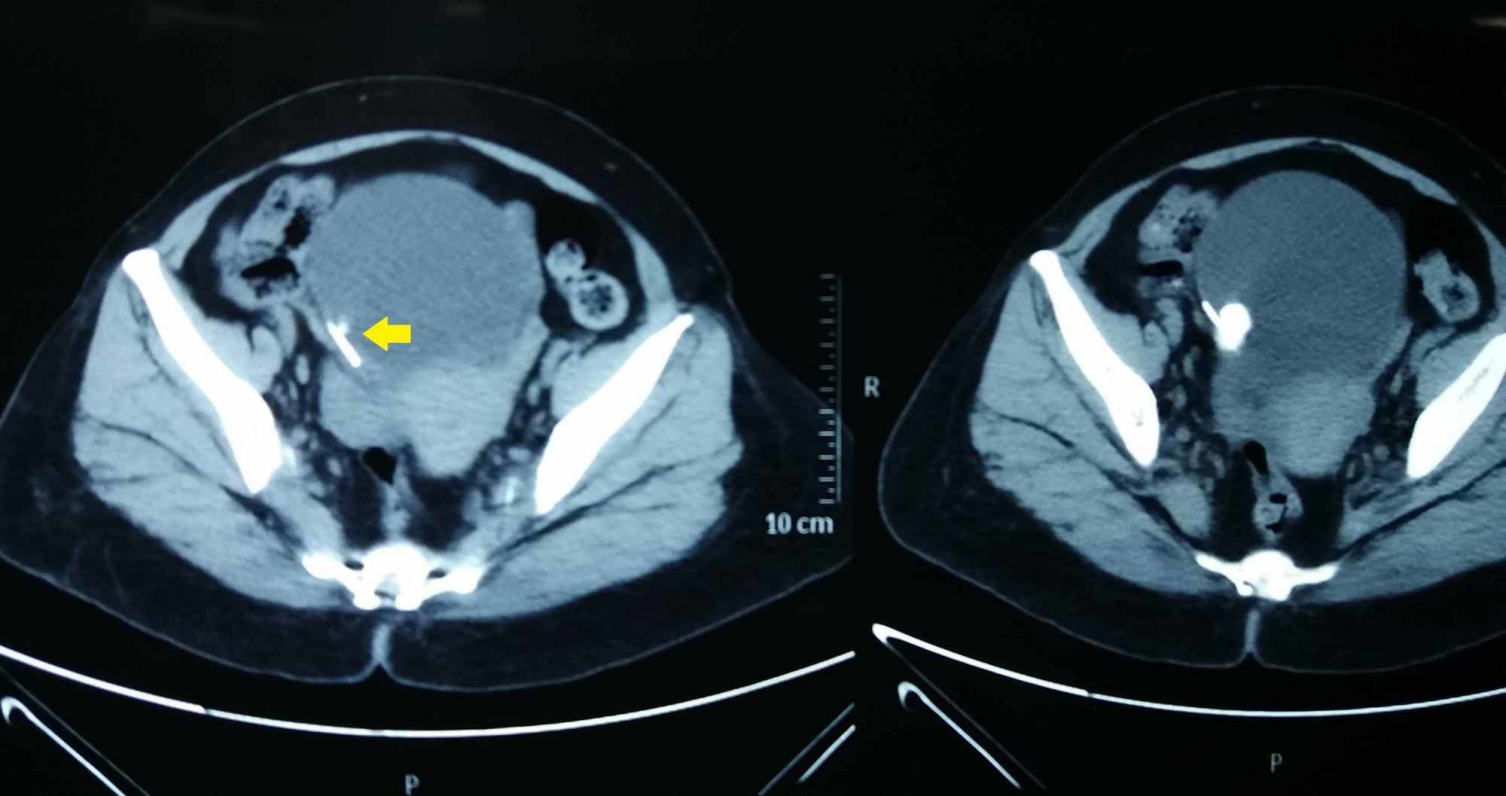
Transverse section of noncontrast computed tomography showing one of the limbs of the copper intrauterine contraceptive device (yellow arrow) embedded within the right lateral bladder wall with overlying calculus

**Figure 3:**
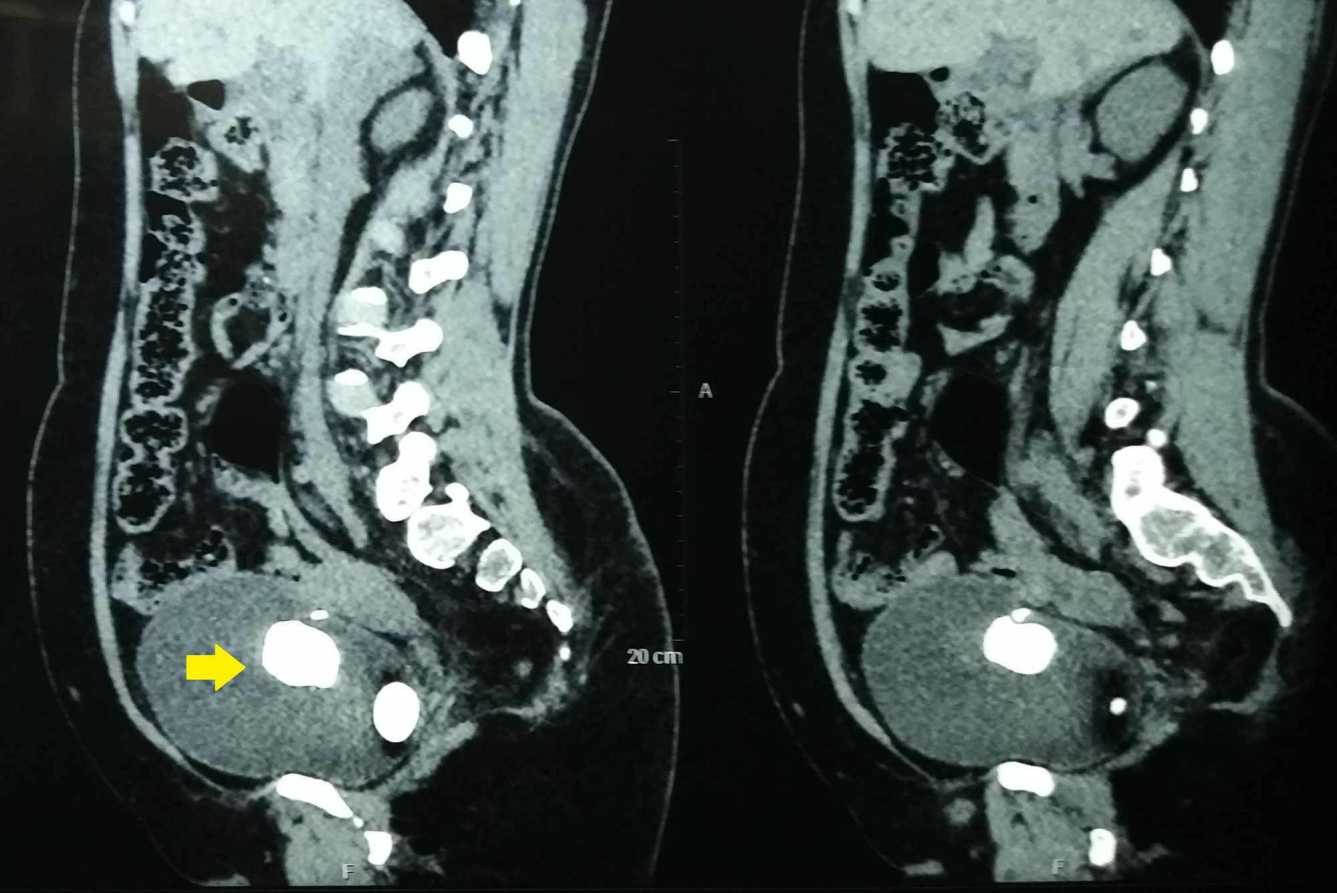
Sagittal section of noncontrast computed tomography depicting two bladder stones, with one hanging from the bladder wall partly embedded within bladder mucosa (yellow arrow)

A 50-year-old woman presented to our outpatient department with a frequency of micturition and dysuria of one-week duration. She denied any other urogynecological symptom or previous urological surgery. She had two children through vaginal deliveries and had not undergone any sterilization procedure in the past. She had no other medical illness, and her general examination was unremarkable. Abdominal and vaginal examinations were normal. Her routine laboratory workup including renal parameters and blood counts were normal, except for pyuria on urinary analysis. A culture of her urine revealed no evidence of infection. However, an ultrasonography of the abdomen and pelvis revealed normal upper tracts with two calculi of 3 cm and 4 cm each in the bladder. Roentgenogram of the KUB (kidney, ureter, and bladder) region revealed two large radio-opaque calculi in the bladder, with clear evidence of one of the stones being formed around a linear radio-opaque intravesical foreign body (Figure 1). On probing the patient further, she recollected having a Cu-IUCD, which was placed immediately after her second vaginal delivery. The thread was not visualized after few weeks of insertion. However, as she was asymptomatic for 25 years, she never bothered to follow up with her gynecologist. Noncontrast computed tomography (NCCT) of the abdomen and pelvis revealed normal upper tract and uterus. However, there were two vesical calculi, one of size 3.5 cm formed over a linear foreign body and hanging from the right lateral wall of the bladder and another of the same size in the dependent portion of the bladder (Figures 2, 3). Surprisingly, there was no evidence of a contraceptive device within the uterus.

**Figure 4:**
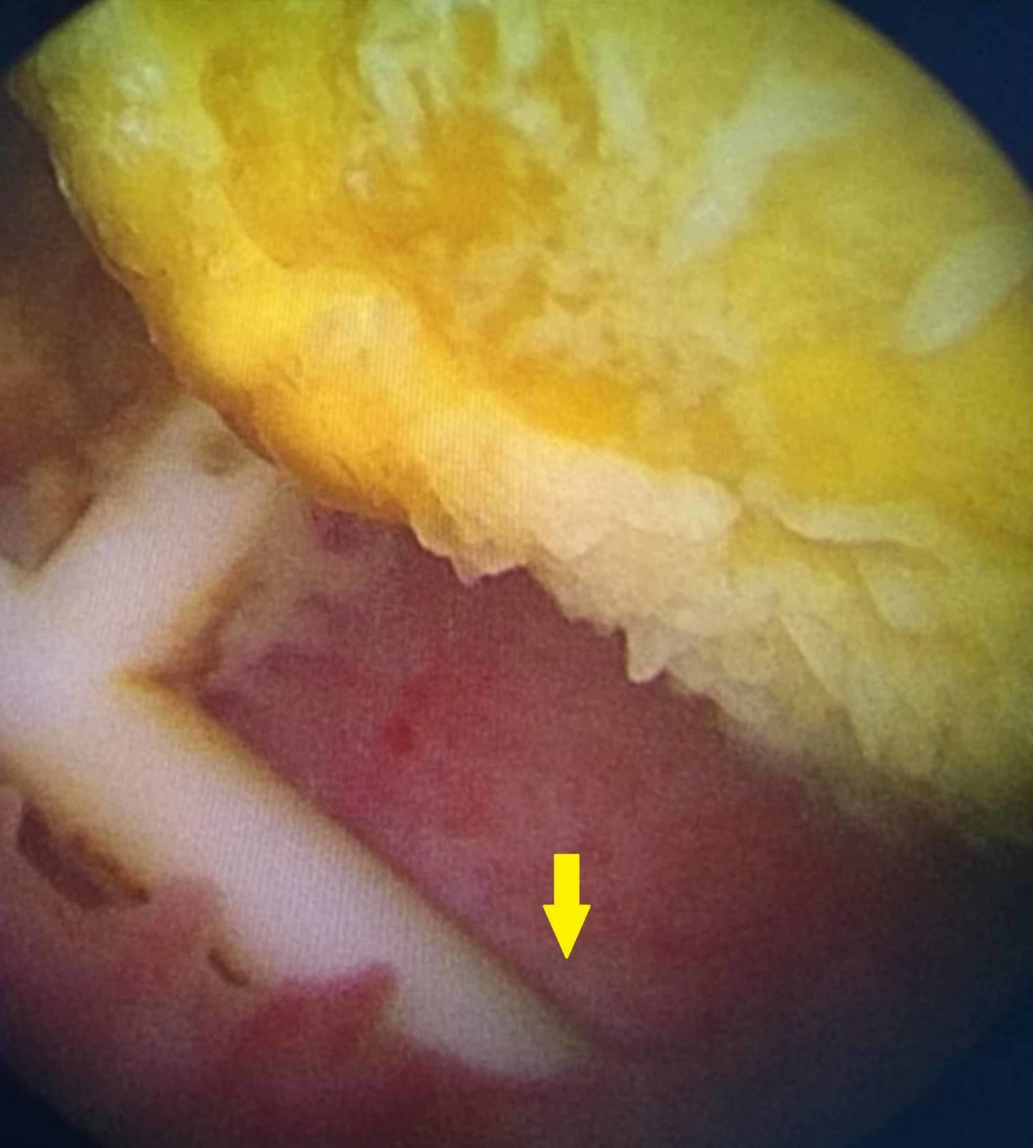
Cystoscopic view of the migrated intrauterine contraceptive device showing the horizontal limb buried within the bladder mucosa (yellow arrow) and stone formation around the vertical limb

**Figure 5:**
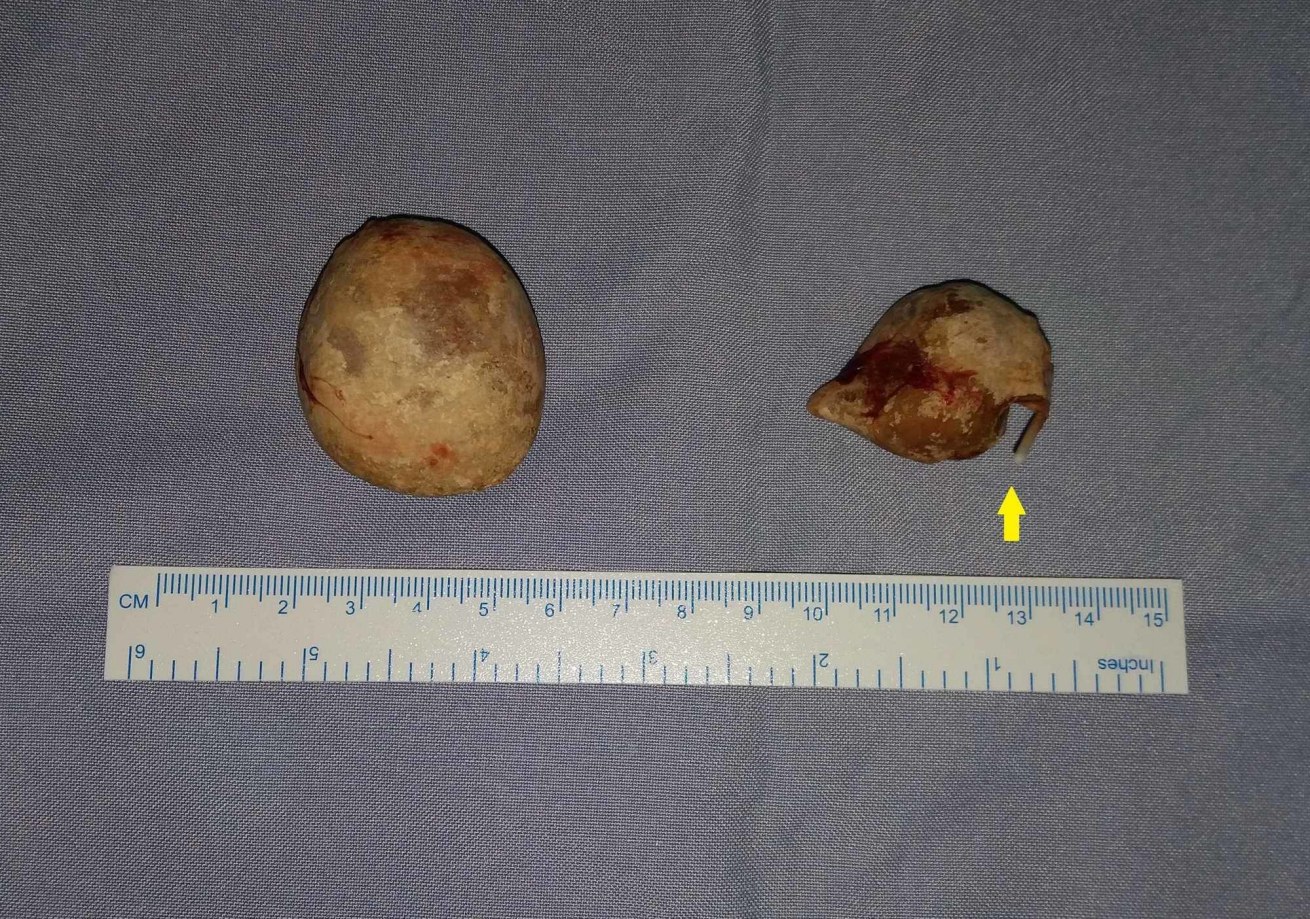
Postoperative image of the vesical calculi formed around the vertical limb of the intrauterine contraceptive device, with the arrow pointing to the horizontal limb

With the clinical suspicion of intravesical migration of IUCD with subsequent stone formation, the patient underwent diagnostic cystoscopy followed by open vesicolithotomy after consenting to the same. Cystoscopy revealed two stones in the bladder. One among them was a freely mobile 3.5-cm vesical calculus, whereas the other similar calculus around the vertical limb of copper-T was hanging from urothelium of the right lateral bladder wall, with horizontal limbs being buried under the urothelium (Figure 4). In view of the large stone size and the possibility of a fistula, open vesicolithotomy was planned. The bladder was approached through a Pfannenstiel incision, and cystotomy was performed, which confirmed the above findings. The hanging vesical calculus was dissected and freed from the mucosa of the bladder, and there was no overt fistula. The calculi were removed followed by closure of the cystotomy, and the patient made an uneventful recovery (Figure 5).

## Discussion

Cu-IUCDs are one of the commonest and cost-effective methods of reversible contraception in developing countries. However, they are fraught with complications such as migration, displacement, infection, extrusion, failure, and dyspareunia. They also increase the risk of abortions and pelvic inflammatory disease.

Uterine perforation and subsequent intravesical migration usually occur by two mechanisms. Firstly, uterine perforation can occur at the time of placement and is characterized by severe pain during insertion of the Cu-IUCD. The second mechanism is due to pressure necrosis of the uterine and bladder walls, which causes erosion and, finally, a fistulous tract between the two organs. However, this is rare, and, most commonly, the Cu-IUCD is found in the peritoneal cavity without penetrating other organs [2]. Several factors such as Cu-IUCD type, anatomic factors, insertion timing, and insertion technique influence the risk of uterine perforation [3]. The normal uterus is anteverted and anteflexed and is closely related to the bladder, which by itself increases the risk of uterine perforation during Cu-IUCD insertion [4]. Similarly, an extreme posterior position of the uterus also favors extrauterine migration of a Cu-IUCD [5].

Commonly, the onset of urinary symptoms can range from three months to five years of Cu-IUCD insertion [4]. While extrauterine migration of Cu-IUCD usually presents with features of pelvic pain, dyspareunia, and chronic pelvic pain, the relatively rare intravesical migration of Cu-IUCD presents with severe LUTSs and recurrent urinary tract infections (UTIs) [6]. Chronic pelvic pain can even contribute to loss of sexual desire and marital disharmony. Thus, Cu-IUCD migration usually presents early due to persistent symptoms. The longest duration of retained Cu-IUCD reported in the literature is 10 years [7]. The present case was surprisingly asymptomatic for 25 years and was detected during the workup for LUTS of one-week duration. The long asymptomatic phase in our patient could be explained by the slow migration and fistulation into the bladder, which occurred over 25 years.

Early diagnosis and treatment of migrated Cu-IUCD is vital to prevent long-term morbidity. Intravesical migration should be suspected in any woman with Cu-IUCD presenting with persistent LUTS or UTI [8]. Ultrasonography and roentgenogram of the KUB region provide a clue to the diagnosis and assessment of the level of encrustation. NCCT is mandatory to better assess the anatomic relationship of the migrated Cu-IUCD with other pelvic organs [4]. In our case, the NCCT scan revealed the exact location of the Cu-IUCD in the pelvis and confirmed the intravesical migration.

All migrated Cu-IUCDs require removal, which can be achieved by different routes. Most of the intravesical Cu-IUCDs can be managed endoscopically [9-10]. Cystoscopy helps in planning the optimal management and must be performed before embarking on removal. Laparoscopy is useful when the Cu-IUCD is extruded into the peritoneal cavity [11]. Cystotomy is required in large stones or associated fistula formation necessitating repair [6]. In our case, the horizontal limbs of Cu-IUCD buried under the urothelium triggered the suspicion of fistulation between the bladder and the uterus, and hence an open cystotomy was performed.

## Conclusions

Persistent LUTS in a woman with Cu-IUCD deserves immediate attention. On the contrary, intravesical migration of Cu-IUCD can be totally asymptomatic for decades, as in our case. A high index of suspicion is mandatory to prevent long-term morbidity and litigations. This case has been presented to highlight the significance of following up even asymptomatic women with IUCDs.
